# Apparent prevalence of *Mycoplasma wenyonii, Candidatus Mycoplasma haemobos*, and bovine leukemia virus in Wisconsin and Michigan dairy cattle herds

**DOI:** 10.3168/jdsc.2020-0033

**Published:** 2021-01-22

**Authors:** R.A. Schambow, K. Poulsen, S. Bolin, D. Krahn, B. Norby, D. Sockett, P.L. Ruegg

**Affiliations:** 1 Center for Animal Health and Food Safety, University of Minnesota School of Veterinary Medicine, St. Paul 55108; 2 Wisconsin Veterinary Diagnostic Lab, Madison 53705; 3 Department of Pathobiology and Diagnostic Investigation, Michigan State University, East Lansing 48824; 4 Country Hills Animals Health Center, Fond du Lac, WI 54937; 5 Department of Large Animal Clinical Sciences, Michigan State University, East Lansing 48824

## Abstract

*Mycoplasma wenyonii* (formerly *Eperythrozoon wenyonii*) is a hemotrophic, epicellular bacterial parasite of cattle that has been associated with clinical disorders, including hemolytic anemia, decreased milk yield, and peripheral edema. *Mycoplasma wenyonii* and a related organism, *Candidatus Mycoplasma haemobos*, have been detected in both ill and apparently healthy cattle, but little is known about their prevalence in US dairy cattle. The objective of this prospective, cross-sectional study was to determine herd-level apparent prevalence of *M. wenyonii* and *C. M. haemobos* in dairy cattle located in Wisconsin and Michigan compared with seroprevalence of bovine leukemia virus (BLV) in the same herds. In summer 2018, researchers collected blood samples from 30 lactating cows per herd from randomly recruited farms in selected dairy-intensive counties in each state. During the farm visit, a brief survey was used to collect herd management information. Detection of *M. wenyonii* and *C. M. haemobos* were based on PCR testing, and ELISA was used to test for antibodies to BLV. Blood samples were collected from lactating cows located in 64 Wisconsin herds (n = 1,930 samples) and 18 Michigan herds (n = 591 samples). Herd-level apparent prevalence was 100% for both *M. wenyonii* and *C. M. haemobos*. Herd-level seroprevalence for BLV was 83 and 100% for Wisconsin and Michigan herds, respectively. Estimated within-herd apparent prevalence of *M. wenyonii* was 71.7% ± 1.0% (ranging from 23.3 to 93.5%) and for *C. M. haemobos* was 77.3% ± 1.0% (ranging from 16.7 to 100%). Within-herd prevalence of BLV positive samples was 39.8% ± 1.0% and ranged from 0 to 86.7%. About 22% of cows were concurrently positive for all 3 organisms. Parity and stage of lactation were recorded for 2,317 cows. Prevalence of positive cows for parity groups 1, 2, and ≥3 were 72.0, 73.8, and 67.7% for *M. wenyonii*; 80.9, 76.8, and 74.9% for *C. M. haemobos*; and 25.3, 39.7, and 55.5% for BLV, respectively. None or only minor differences in apparent prevalence were observed based on stage of lactation. This is the first report of the prevalence of hemotrophic mycoplasmas in Wisconsin and Michigan dairy herds and indicates that infection with these organisms is endemic. The impact of infection on cattle health and productivity remains unknown, and risk factors associated with infection warrant further study.

## Highlights

• Blood was collected from 1,930 (64) and 591 (18) dairy cows (farms) in Wisconsin and Michigan
• >70% of sampled cows and .5 cows in all herds tested positive for *Mycoplasma wenyonii* or *Candidatus Mycoplasma haemobos*
• All Michigan and >83% of Wisconsin herds had .1 cow test positive for bovine leukemia virus (BLV)
• Overall prevalence of BLV antibodies was ~40%
• Exposure to these organisms was widespread and the effect of infections on animal health and productivity is unknown

## Graphical Abstract

**Figure:**
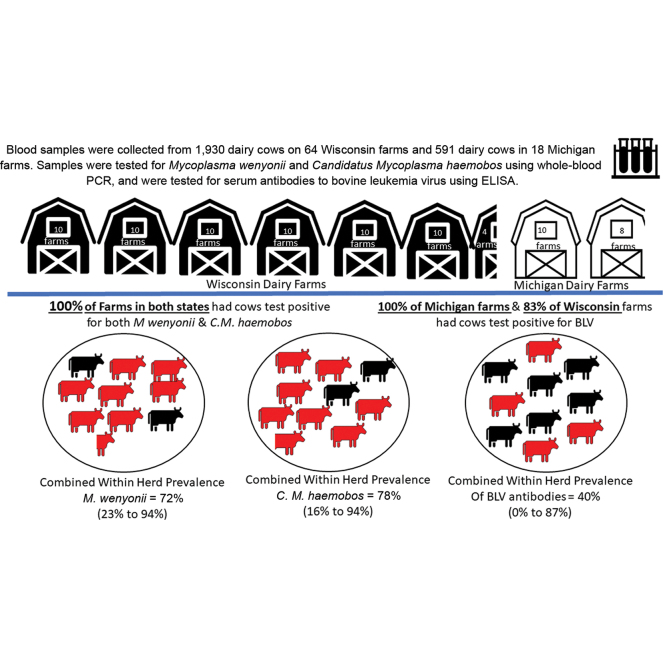
**Summary:***Mycoplasma wenyonii* and a related organism, *Candidatus Mycoplasma haemobos*, have been detected in both ill and apparently healthy cattle, but little is known about their prevalence in US dairy cattle. Blood samples were collected from 1,930 dairy cows on 64 Wisconsin farms and 591 dairy cows in 18 Michigan dairy farms. Samples were tested for *Mycoplasma wenyonii* and *Candidatus Mycoplasma haemobos* using whole-blood PCR, and were tested for serum antibodies to bovine leukemia virus using ELISA. This is the first report of the prevalence of hemotrophic mycoplasmas in Wisconsin and Michigan dairy herds and indicates that infection with these organisms is endemic.

Hemotrophic mycoplasmas (hemoplasmas) lack a cell wall and have a tropism for mammalian red blood cells. They have been found in many species, including dogs and cats (Messick, 2003), swine (Gatto et al., 2019), small ruminants (Aktas and Ozubek, 2017), reindeer (Stoffregen et al., 2006) and cattle (Strugnell and McAuliffe, 2012; de Mello et al., 2019). Hemoplasma infections have been found in healthy animals as well as being associated with a range of clinical signs, including chronic subclinical to life-threatening hemolytic anemia (Genova et al., 2011; McFadden et al., 2016), production loss (Tagawa et al., 2013), and infertility (Montes et al., 1994), depending on the species of hemoplasma and immune state of the individual (Strugnell and McAuliffe, 2012). Researchers have proposed that these organisms may even be important cofactors in the development of secondary infections and neoplastic diseases, such as in cats infected concurrently with *Haemobartonella felis* (now named *Mycoplasma hemofelis*) and feline leukemia virus (Bobade et al., 1988).

*Mycoplasma wenyonii*, formerly known as *Eperythrozoon wenyonii* (Neimark et al., 2002), and *Candidatus Mycoplasma haemobos* are both hemotrophic, epicellular bacterial parasites. *Mycoplasma wenyonii* was first identified in a splenectomized calf in 1934 (Adler and Ellenbogen, 1934) and has since been identified in several countries (dos Santos et al., 2012; McFadden et al., 2016; Quiroz-Castañeda et al., 2018; Díaz-Sánchez et al., 2019; Nouvel et al., 2019). Based on phylogenetic evidence and its 16S ribosomal RNA gene sequence, it was recently reclassified into the *Mycoplasma* genus (Ayling et al., 2012). Various clinical signs have been associated with *M. wenyonii* infection in cattle, including hemolytic anemia, pyrexia, infertility, decreased milk production, prefemoral lymphadenopathy, rough haircoat, weight loss, and scrotal, teat, and hind limb edema (Strugnell and McAuliffe, 2012). Chronic infection with *M. wenyonii* or *C. M. haemobos*, or co-infection have been associated with decreased milk production (Tagawa et al., 2013). Diagnosis was traditionally made via microscopic visualization of organisms on acridine orange- or Giemsa-stained blood smears, but these methods are of low sensitivity and specificity for hemotrophic bacterium (Ritzmann et al., 2009), and PCR is now the preferred method of diagnosis (Hoelzle et al., 2011). When clinical signs are present, treatment with oxytetracycline has reportedly resulted in resolution of signs in some animals (Montes et al., 1994; Genova et al., 2011). Hemotrophs are transmitted via blood, and arthropod vectors and direct contact of blood via fomites have been suggested as possible sources (Smith et al., 1990). Vertical transmission has also been shown, but its overall significance as a route of transmission remains unknown (Hornok et al., 2011; Sasaoka et al., 2015; Niethammer et al., 2018).

Hemoplasmas have been identified in apparently healthy cattle (Strugnell and McAuliffe, 2012), but the clinical relevance of subclinical infection with *M. wenyonii* and *C. M. haemobos* remains largely unknown. This study was undertaken to estimate prevalence of *M. wenyonii* and *C. M. haemobos* via PCR compared with seroprevalence of bovine leukemia virus (**BLV**) in the same herds and to generate preliminary data to identify potential risk factors (including within-herd prevalence of BLV) for infection and transmission in dairy cattle herds in selected regions of Wisconsin and Michigan.

The study was designed as a prospective, cross-sectional study, with herd as the primary experimental unit. Herds in selected dairy-intensive counties in Michigan and Wisconsin containing a minimum of 50 lactating dairy cows were eligible for inclusion in the study. Selection of herds was stratified by herd size to reflect the herd size distribution in the Upper Midwest region, where approximately 60% of eligible herds contain ≥200 cows (larger herds) and 40% of eligible herds contain <200 cows (small herds) (https://quickstats.nass.usda.gov/). Herds were initially selected using a random number generator from among all herds on the dairy permit and confined animal feeding operation permit lists of the top 10 Wisconsin dairy cow counties (based on number of dairy cows), and then mailed a letter containing a prepaid return postcard informing them of the study. In Michigan, randomly selected herds in 10 counties were mailed a recruitment letter using a similar process. Producers who returned the postcard indicating willingness to participate were contacted by phone to schedule a sampling visit. Within-herd, a sample size of 30 cows per herd was calculated to detect ≥10% prevalence of infected cows with 95% confidence.

Each herd was visited once for sampling between July and August 2018. All lactating cows were eligible for sampling, regardless of breed, parity, production, stage of lactation, or reproductive status. To alleviate producer concerns about time demands related to searching for randomly selected cows on very large dairy farms, during the farm visit, researchers selected a convenience sample of healthy cows that were distributed among all cattle pens and age groups. Study personnel collected whole blood samples from the coccygeal vessels of about 30 cows using 10-mL serum-separator and EDTA tubes. After sampling, parity, and stage of lactation for each sampled cow were collected from herd records. Blood samples were immediately cooled to 4°C for transport to the Michigan State University Veterinary Diagnostic Laboratory (Lansing, MI) for processing.

Immediately following sample collection, a brief questionnaire (available from the authors) was given to an owner or manager of each farm with the purpose of identifying preliminary data about potential risk factors related to the epidemiology of *M. wenyonii, C. M. haemobos*, and BLV. General questions were asked about housing, feeding, and husbandry, vaccination and treatment protocols, and reproductive management. This study was approved by the Institutional Animal care and Use Committee at Michigan State University (09-17-155-00) and was deemed exempt from human subjects requirements by the Institutional Review Board at Michigan State University (IRB x17–1538e).

Whole blood harvested into evacuated tubes containing EDTA was used for DNA extraction. A commercial kit (Quick-DNA Miniprep Plus Kit, Zymo Research, Irvine, CA) was used to extract DNA from 200 µL of blood following kit instructions. The kit uses a silica-based DNA purification method, and the purified DNA was eluted with 75 µL of elution buffer supplied with the kit. The eluted DNA was used in a PCR assay targeting the 16S ribosomal RNA gene of *M. wenyonii* and *C. M. haemobos*. The amplification products differed by 13 bp, which was sufficient to allow detection of mixed infections and identification of individual hemoplasma (Jensen et al., 2001; Tagawa et al., 2008). The PCR reaction used AmpliTaq Gold 360 Master Mix (Applied Biosystems, Foster City, CA). The reaction conditions were 95°C for 5 min followed by 40 cycles of 95°C for 30 s, 56°C for 30 s, and 72°C for 30 s, followed by 72°C for 5 min. An agarose-based electrophoresis system and ethidium bromide staining was used for detection of amplified product. Representative PCR products, at least 3 from each herd, were processed using ExoSAP-IT Express (Life Technologies Corp., Carlsbad, CA) and submitted to the Research Technology Support Facility at Michigan State University for Sanger sequencing to verify identity. To reduce risk of cross-contamination during the PCR assays, reagent preparation, nucleic acid extraction, addition of sample DNA to the PCR reaction tubes, and the PCR assay all were done in separate rooms with dedicated supplies and laboratory gowns.

Detection of antibody against the bovine leukemia virus viral glycoprotein 51 (gp51) was done using commercially available ELISA kit (Bovine Leukemia Virus Antibody Test Kit, VMRD Inc., Pullman, WA) following the manufacturer's recommendations for performance and interpretation of results.

Descriptive data were reviewed and evaluated for normality, and associations between herd characteristics and state were determined using either ANOVA (continuous variables) or Chi-squared tests (categorical variables). Within-herd apparent prevalence was calculated for each state and for the combined data. Both rank-sum and *t*-tests were used to compare prevalence of organisms (*M. wenyonii* or *C. M. haemobos*) and antibodies (BLV) between states. After determining that the organisms were endemic, data from all herds was combined, and cow-level data were used as the experimental unit to test associations between prevalence of organisms or antibodies and parity or stage of lactation. Parity was categorized into 3 groups: parity 1, 2 and ≥3, and stage of lactation was categorized as 0 to 68, 69 to 140, 141 to 239, and ≥240 DIM. Associations between parity group or stage of lactation group and prevalence of organisms were evaluated by Bartlett's test for equal variances, and separation among least squares means was performed using Bonferroni tests. Associations between apparent prevalence and selected risk factors were evaluated using ANOVA. Statistical analysis was performed using SAS v9.4 (SAS Institute Inc., Cary, NC); statistical significance was declared at *P* < 0.05 and trends were defined at *P* < 0.10.

Recruitment letters were sent to 375 of about 3,000 dairy farms located in the 10 selected dairy-intensive counties in Wisconsin, and 113 producers (30.1%) returned postcards indicating that they were interested in participating. Ultimately, 64 Wisconsin farms were enrolled based on ease of scheduling visits. In Michigan, letters were sent to 120 of about 600 dairy farms located in 10 counties, and 18 farms (15.0%) responded affirmatively and were enrolled. Enrolled herds together contained about 88,133 lactating and dry cows, 9,554 preweaning calves, and 40,363 replacement heifers. The median number of mature cows per herd was 350, but herds ranged in size from 56 to 8,833 mature cows (mean = 1,075 ± 176). Most management practices and farm characteristics did not vary by state (*P* ≥ 0.10) but Wisconsin herds contained fewer purchased cattle (2.4% of Wisconsin cows and 8.8% of Michigan cows; *P* = 0.003) and slightly more primiparous cows (31.1% for Michigan and 37.5% for Wisconsin herds; *P* = 0.011). Typical of dairy farms in the Upper Midwest, cows were primarily housed in freestalls (81% of farms), but tiestalls were used on 17% of farms (all in Wisconsin), and 2 Wisconsin farms used loose housing for some adult cows. About 36% of farms gave some groups of cattle at least occasional access to pasture. Almost all cows were fed a TMR and 57% of farms milked cows at least 3 times per day. Fly control was reported to be used extensively for both calves and cows. Calf housing was more variable than housing of adult cows and included outdoor hutches (40%), individual pens in indoor calf barns (25%), indoor group pens (14%), and combinations of the former (22%). Based on estimates provided during the survey, beginning at birth, the typical mature cow had received a total of 65.4 ± 5.7 injections (inclusive of vaccines, oxytocin, supplements, and reproductive hormones but not treatments). Almost 80% of interviewees reported that they used needles on more than one animal and estimated that the average needle was used for 15.1 ± 2.6 injections. Similarly, most farmers reported using palpation sleeves on multiple animals.

Blood samples (n = 2,521) were collected from mature cows located in Wisconsin herds (n = 1,930 samples; median of 30.0 per farm; ranging from 30 to 41) and Michigan herds (n = 591 samples; median of 30.5 samples per farm; ranging from 30 to 32). *Mycoplasma wenyonii* and *C. M. haemobos* were identified by PCR in blood samples obtained from at least 5 cows in all herds (herd-level apparent prevalence of 100%). Overall, within-herd apparent prevalence of *M. wenyonii* was (mean ± SE) 71.7% ± 1.0% and ranged from 23.3 to 93.5%. Overall apparent prevalence of *C. M. haemobos* was 77.3% ± 1.0% and ranged from 16.7 to 100%. At least 1 BLV positive blood sample was found in 83% of Wisconsin herds and 100% of Michigan herds. Overall prevalence of BLV antibodies was 39.8% ± 1.0% and ranged from 0 to 86.7%. Within-herd apparent prevalence of *M. wenyonii* was greater for cows sampled in Michigan herds (76.5% ± 1.7%) than in Wisconsin herds (70.1% ± 1.0%; *P* = 0.003). In contrast, within-herd apparent prevalence of *C. M. haemobos* did not vary between states and was 78.9% ± 1.7% and 76.8% ± 1.0% for cows sampled in Michigan and Wisconsin, respectively (*P* = 0.30). Likewise, within-herd apparent prevalence of BLV did not vary between states and was 41.5% ± 2.0% and 39.3% ± 1.1% for cows in Michigan and Wisconsin herds, respectively (*P* = 0.36). About 22% of cows were concurrently positive for both organisms and BLV antibodies, and this prevalence did not vary by state (*P* = 0.49)

Parity was recorded for 807, 696, and 814 cows in parities 1, 2, and ≥3, respectively (Figure 1). The apparent prevalence of cows positive for *M. wenyonii* was less for cows in parity group ≥3 compared with that in primiparous cows (*P* = 0.03) but did not differ between primiparous cows and cows in their second lactation (*P* > 0.16). Similarly, apparent prevalence of cows positive for *C. M. haemobos* was less for cows in parity group ≥3 compared with other parity groups (*P* = 0.01) but did not differ between parity groups 1 and 2 (*P* > 0.16). As previously reported (Erskine et al., 2012), prevalence of BLV-positive blood samples increased with parity group (Figure 1, *P* < 0.001).

**Figure 1:**
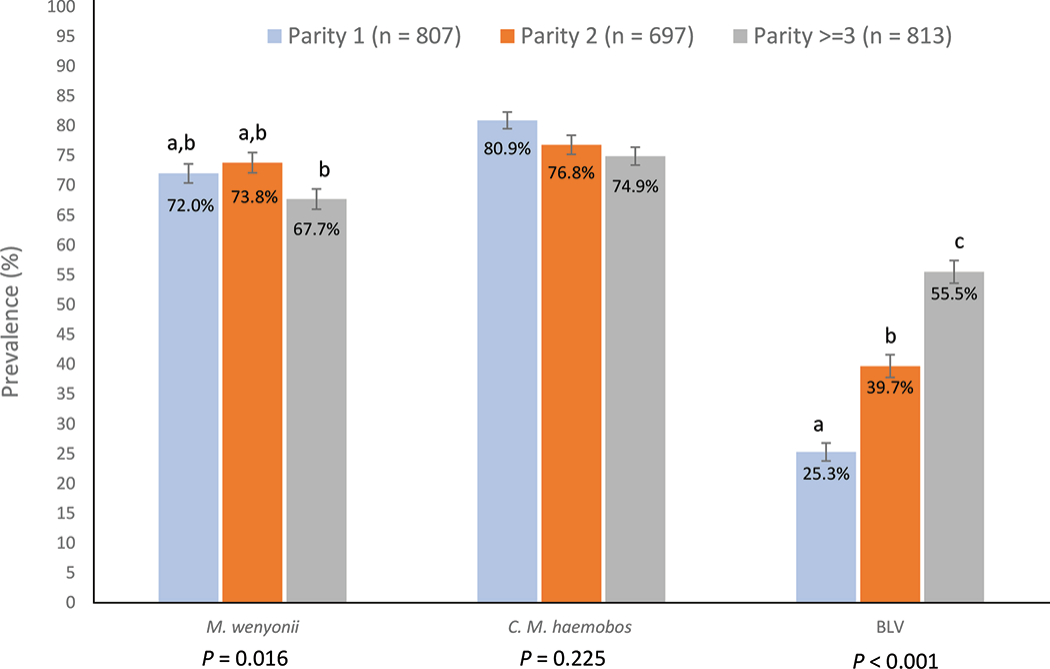
Prevalence of positive hemotrophic mycoplasma PCR tests for *Mycoplasma wenyonii* and *Candidatus Mycoplasma haemobos* and serum bovine leukemia virus (BLV) antibody tests by parity for dairy cows sampled in Wisconsin (n = 64) and Michigan (n = 18) herds. Differing letters (a–c) denote significant differences among parities within organism; SE denoted by error bars. *P*-value derived from Bartlett's test of equal variances.

Stage of lactation (DIM) was recorded for 1,970 lactating cows and dry cows and ranged from 0 (23 dry cows) to 682 d (Figure 2). There was no association between stage of lactation and apparent prevalence of *M. wenyonii* (*P* = 0.40) or apparent prevalence of BLV antibodies (*P* = 0.86; Figure 2). The apparent prevalence of *C. M. haemobos* was less for cows in early lactation (70.0% for 0 – 68 DIM) than for cows in all other stages of lactation (80.0, 82.4, and 78.1% for 69–140 141–239, and >240 DIM, respectively, *P* < 0.001; Figure 2).

**Figure 2:**
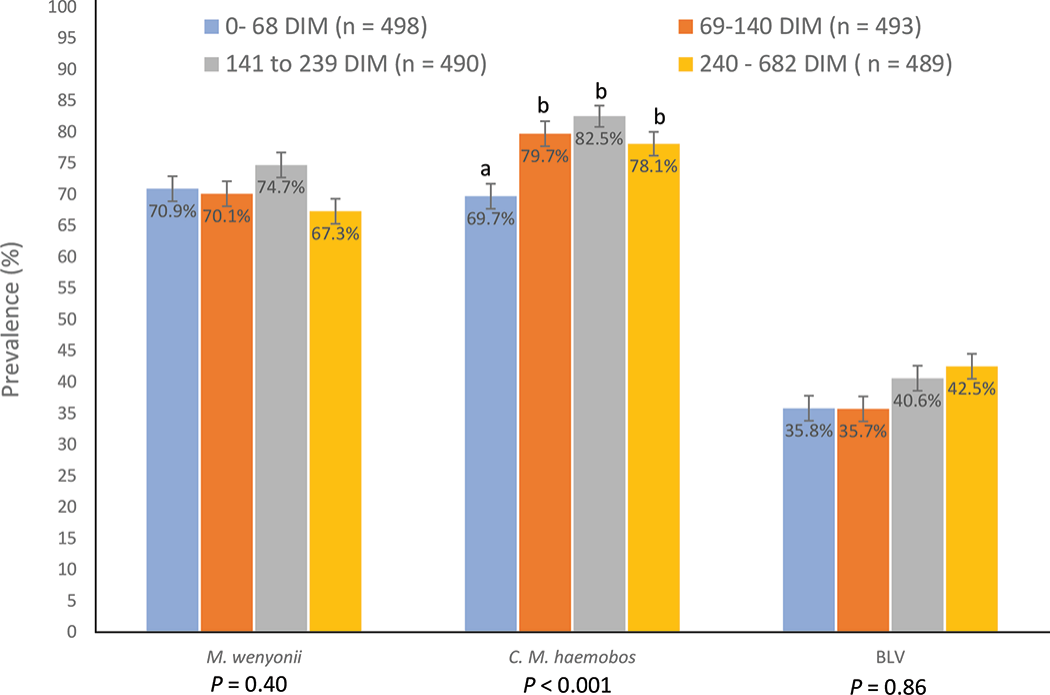
Prevalence of positive hemotrophic mycoplasma PCR tests for *Mycoplasma wenyonii* and *Candidatus Mycoplasma haemobos* and serum bovine leukemia virus (BLV) antibody tests by stage of lactation for dairy cows sampled in Wisconsin (n = 64) and Michigan (n = 18) herds. Differing letters (a, b) denote significant differences among stage of lactation groups within organism**;** SE denoted by error bars. *P*-value derived from Bartlett's test of equal variances.

Although this was an exploratory study with the primary objective of determining prevalence of hemotrophic mycoplasma organisms, to provide direction for future studies with more herds we performed a preliminary bivariate analysis to determine relationships between putative risk factors and apparent prevalence of hemotrophic mycoplasma and BLV (Table 1). No associations were seen between apparent prevalence of any organism and herd-size category or use of rectal sleeves on >1 cow. Among organisms, there was a tendency for lower within-herd apparent prevalence only of *M. wenyonii* for herds that did not allow access of any cattle to pasture (*P* = 0.09) and for herds that reported purchase of ≥1 animals (Table 1; *P* = 0.07). Within-herd apparent prevalence of BLV tended to be greater for herds that reported using >1 needle per cow (*P* = 0.08). Within-herd apparent prevalence was greater for both *M. wenyonii* and *C. M. haemobos* when cattle were housed in herds that contained BLV-positive animals, but all BLV-negative herds were located in Wisconsin, so that association is confounded by state (Table 1; *P* < 0.05). We identified an interesting potential association of within-herd apparent prevalence and calf housing (Table 1). Cows in herds that used only indoor individual calf housing had a lower apparent prevalence of *C. M. haemobos* than herds that used outdoor hutches only (Table 1). Although this association was not statistically significant for other organisms (or BLV antibodies), the same numerical trend was seen, indicating that further investigation of the role of outdoor calf housing and infection with blood borne agents should be considered in studies that include more herds and have greater statistical power. It is important to note that this study was not designed to have sufficient power for a risk factor analysis, so potential risk factors noted in this study warrant further investigation.

**Table 1:** Apparent prevalence of hemotrophic mycoplasmas^1^ and bovine leukemia virus (BLV) serum antibodies for selected herd characteristics

Risk factor	Within-herd apparent prevalence, % (SE)			
	*M. wenyonii*	*C. M. haemobos*	BLV	All 3 organisms
Herd size (no. of milking and dry cows)				
<250 cows (n = 35)	0.72 (0.02)	0.77 (0.02)	0.37 (0.04)	0.21 (0.03)
251–1,000 (n = 20)	0.73 (0.03)	0.76 (0.03)	0.38 (0.05)	0.21 (0.04)
>1,000 (n = 27)	0.70 (0.02)	0.79 (0.02)	0.46 (0.04)	0.24 (0.03)
*P*-value among categories	0.78	0.67	0.26	0.79
Animals on pasture				
Yes (n = 29)	0.75 (0.02)	0.77 (0.02)	0.41 (0.04)	0.25 (0.03)
No (n = 51)	0.69 (0.03)	0.78 (0.02)	0.39 (0.03)	0.20 (0.02)
*P*-value among categories	0.09	0.67	0.65	0.15
Closed herd				
Yes (n = 36)	0.75 (0.02)	0.76 (0.02)	0.41 (0.04)	0.23 (0.03)
No (n = 46)	0.69 (0.02)	0.78 (0.02)	0.39 (0.03)	0.21 (0.02)
*P*-value among categories	0.07	0.38	0.72	0.61
Cows palpated per rectal sleeve				
1 (n = 28)	0.73 (0.03)	0.74 (0.02)	0.37 (0.04)	0.21 (0.03)
>1 cow (n = 54)	0.71 (0.02)	0.79 (0.02)	0.42 (0.03)	0.23 (0.02)
*P-*value among categories	0.66	0.14	0.34	0.50
Cows injected per needle				
1 (n = 17)	0.71 (0.04)	0.80 (0.03)	0.31 (0.05)	0.18 (0.04)
>1 cow (n = 65)	0.72 (0.02)	0.77 (0.02)	0.42 (0.03)	0.23 (0.02)
*P*-value among categories	0.89	0.32	0.08	0.24
BLV-positive herd				
Yes (n = 71)	0.74 (0.02)	0.78 (0.04)		
No (n = 11)	0.58 (0.04)	0.70 (0.02)		
*P-*value among categories	0.001	0.05		
Calf housing				
Outdoor hutch (n = 32)	0.74 (0.03)	0.81^a^ (0.02)	0.45 (0.04)	0.26 (0.03)
Indoor individual (n = 20)	0.65 (0.03)	0.70^b^ (0.03)	0.34 (0.05)	0.17 (0.04)
Indoor group (n = 11)	0.76 (0.05)	0.78^ab^ (0.04)	0.49 (0.07)	0.29 (0.05)
Other or various (n = 18)	0.72 (0.04)	0.79^ab^ (0.03)	0.33 (0.05)	0.18 (0.04)
*P*-value among categories	0.13	0.01	0.10	0.10

a,b Means with differing letters indicate significant differences among categories within agent. *P*-values were derived from ANOVA and corrected using Bonferroni tests.

1 *Mycoplasma wenyonii*and *Candidatus Mycoplasma haemobos*.

To our knowledge, this is the first prevalence study of hemotrophic mycoplasmas in dairy herds located Wisconsin and Michigan, and it indicates that infection is endemic in these 2 states. The endemic nature of the infections and high within-herd prevalence were unexpected, and more research is urgently needed to identify the effect of these infections and the risk factors associated with infection. Herds that participated in this study volunteered after randomly receiving recruitment letters, so it is possible that selection bias may have influenced our results, but the 100% herd-level prevalence is highly suggestive that infection with these organisms is widely distributed in these northern states. The impact of infection with hemoplasmas on health, well-being, and productivity of dairy cows is not well defined. Hemoplasmas have been associated with several vague clinical syndromes of cattle in several countries (Tagawa et al., 2008; Genova et al., 2011; Hoelzle et al., 2011; Strugnell et al., 2011; Yuan et al., 2011; Ayling et al., 2012; Gladden et al., 2016). Clinical signs have included immune-mediated anemia, edema of the mammary gland and rear legs, pyrexia, lymphadenopathy, reduced milk yield, weight loss, and infertility (Smith et al., 1990; Montes et al., 1994; Scott, 2008; Genova et al., 2011; Hoelzle et al., 2011; Strugnell et al., 2011; Gladden et al., 2016). In the United States, the first apparent case of *Eperythrozoon wenyonii* was reported in Colorado in 1990, using blood smears to diagnose the disease in a group of primiparous dairy cows in a large dairy farm (Smith et al., 1990). The animals were reported to have edema in the teats and distal limbs, prefemoral lymphadenopathy, fever, reduced milk yield, weight loss, and reproductive inefficiency. In 1994, a young Charolais bull in Oklahoma was diagnosed with transient infertility after experiencing clinical signs of scrotal and hindlimb edema accompanied by fever, lethargy, and anemia (Montes et al., 1994). The diagnosis was made based on observation of large numbers of *E. wenyonii* on blood smears. Similar signs of disease have been associated with *C. M. haemobos* and co-infection with both *M. wenyonii* and *C. M. haemobos* (Tagawa et al., 2008; Hoelzle et al., 2011). Chronic subclinical infection with these organisms has been associated with reduced milk yield (Tagawa et al., 2013). Although infection with these organisms has been demonstrated to be associated with occurrence of specific clinical signs, the organisms have also been identified in blood of apparently healthy animals (Strugnell et al., 2011; Yuan et al., 2011; McFadden et al., 2016), and research on the duration and impact of infection in dairy cows is needed.

Our data did not demonstrate consistent trends associating apparent prevalence with parity or stage of lactation, but the high prevalence in primiparous cows and lack of increasing prevalence with stage of lactation suggest that infection may have occurred before initiating lactation. While prevalence of hemotrophic mycoplasma was the subject of our research, we evaluated seroprevalence of BLV because we hypothesized that transmission mechanisms may be similar, and understanding BLV prevalence may help identify potential risk factors for further study. However, in contrast to the typical increased prevalence of BLV as cows age, we observed slightly lower apparent prevalence for *M. wenyonii* only in parity group ≥3. Apparent prevalence of BLV increased with parity but was significantly lower than that of the hemotrophs. We detected hemoplasmas using PCR and detected the presence of BLV based on serum antibodies, so comparisons should be made very cautiously. We hypothesize that the transmission mechanisms should be similar, but it is possible that clearance of hemoplasma is enhanced with age or that the concentration of hemoplasma organisms decreases with age, thus reducing the sensitivity of detection. Future studies should include sampling of youngstock beginning at birth and, to discern the role of vertical transmission, should include dam and calf pairs. Given the endemic nature of infection and high within-herd prevalence in both states, longitudinal studies are needed to identify the role of calf housing, flies, and other potential vectors.

Transmission of hemoplasmas is through contact with blood, but the ability of other bodily fluids to infect susceptible hosts is not known (Strugnell and McAuliffe, 2012). Bloodborne transmission is thought to occur primarily through use of common needles, rectal palpation sleeves, and other fomites such as tools used for dehorning and hoof-trimming (Strugnell and McAuliffe, 2012). Our small study was not able to confirm associations between the use of common needles or palpation sleeves; larger studies that include herds with lower apparent prevalence are needed to better define the risk factors for infection. Authors of a case report suggested that the incidence of positive animals was greater in summer and fall and suggested that transmission by arthropod vectors may have been responsible, but they could not confirm a causal relationship (Strugnell and McAuliffe, 2012). Given that all herds enrolled in this study were in northern regions, transmission by arthropod vectors would be unlikely for at least 3 to 4 mo of the year, restricting the ability of these insects to consistently contribute to transmission. Although lice (Hofmann-Lehmann et al., 2004), house flies, stable flies, and horn flies (Hornok et al., 2011) have all been demonstrated to be capable of infection and mechanical transmission, the ability of blood-sucking insects to serve as reservoirs is unknown (Hornok et al., 2011). Vertical transmission has been demonstrated to occur for both *M. wenyonii* and *C. M. haemobos*, but the clinical significance of fetal infection is not known (Sasaoka et al., 2015). Infection with these organisms is increasingly being recognized by dairy practitioners in Michigan and Wisconsin, and broader studies are needed as a first step in evaluating the risk of this disease to health and productivity of dairy cattle.
